# Comparative Genomic Analysis of *Xanthomonas campestris* pv. *campestris* Isolates BJSJQ20200612 and GSXT20191014 Provides Novel Insights Into Their Genetic Variability and Virulence

**DOI:** 10.3389/fmicb.2022.833318

**Published:** 2022-03-02

**Authors:** Denghui Chen, Xionghui Zhong, Jian Cui, Hailong Li, Rui Han, Xiangqing Yue, Jianming Xie, Jungen Kang

**Affiliations:** ^1^College of Horticulture, Gansu Agricultural University, Lanzhou, China; ^2^Beijing Vegetable Research Center, Beijing Academy of Agriculture and Forestry Sciences, Key Laboratory of Biology and Genetic Improvement of Horticultural Crops (North China), Ministry of Agriculture, Beijing, China

**Keywords:** brassica oleracea, black rot, virulence, genomic comparisons, *Xanthomonas campestris* pv. *campestris*, IS family transposase, whole-genome sequencing, effectors

## Abstract

Black rot is a disease that has a severe impact on cabbage yield and quality in China. *Xanthomonas campestris* pv. *campestris* (*Xcc*) is the causal agent of black rot of Brassicaceae crops. So far, the whole genomic sequences of more than 30 *Xcc* isolates have been sequenced; however, little information about genomic variability and virulence has been reported. In this study, 12 *Xcc* isolates were isolated from diseased cabbage leaves in seven Chinese provinces and two municipalities from July 2019 to November 2020. Pathogenicity analysis showed that isolate GSXT20191014 was more aggressive than BJSJQ20200612 and HRIW 3811 on cabbage inbred line 1371. Both BJSJQ20200612 and GSXT20191014 were sequenced and comparatively analyzed. The results showed that BJSJQ20200612 and GSXT20191014 have a single circular chromosome comprising 5,115,975 and 4,975,682 bp, respectively. Compared to the other six sequenced strains, 26 and 47 variable genomic regions were found in BJSJQ2020061 and GSXT20191014 genomic sequences, respectively. The variable genomic regions could be responsible for the genetic variation in *Xcc* strains and have led to the differences in type III secreted effector repertoires, virulence factors and secreted proteins between these two strains. Among the identified secreted proteins, two copies of peptidase S8/S53 were found in GSXT20191014-specific chromosomal segments. The common effectors *xopR*, *xopH*, *avrBs1*, and *xopAH* are found in most *Xcc* genomes, but they are absent in the GSXT20191014 genome. Variations in the composition of exopolysaccharides (EPS) and lipopolysaccharides (LPS) may aid GSXT20191014 isolate infections to evade recognition by the host immune system. Our results revealed a direct correlation between genomic variability and *Xcc* virulence. We also developed several markers for detecting BJSJQ20200612 and GSXT20191014 isolates and further tested the rest of our other 10 isolates. Finally, the isolated *Xcc* strains were classified into three genetic subgroups by specific molecular markers and multilocus sequence typing (MLST) approach. BJSJQ20200612 and GSXT20191014 isolates were also classified into two subgroups of *Xcc* according to the core-genome-based phylogenetic tree. This study extended our understanding of *Xcc* genomic features and provided the foundation to further characterize the mechanisms for *Xcc* virulence and a clue for black rot resistance breeding.

## Introduction

Black rot of crucifers, caused by *Xanthomonas campestris* pv. *campestris* (*Xcc*), was first reported as a cabbage disease by Garman in 1890 in Kentucky, United States (Garman, 1890). Since then, the disease has been reported in all *Brassicaceae*-growing regions, and it is considered as one of the most destructive diseases of crucifers worldwide (Williams, 1980).

Black rot is a seed-borne disease. *Xcc* is favored by warm and humid conditions and *Xcc* can survive overwinter on plant debris, crucifer weeds, and wild relatives of cultivated brassicas (Cook et al., 1952; Schaad, 1981). These bacteria establish a systemic infection in susceptible hosts by penetrating the plant through the hydathodes at leaf margins or injuries. The typical symptom of black rot is the formation of V-shaped chlorotic yellow lesions along the leaf margins. Furthermore, the darkened veins appear along with the bacterial movement in the vascular system. Eventually, the affected tissues become necrotic and leaves fall prematurely, systemic infections cause stunted growth and the death of young plants. Black rot disease drastically reduces crop yields during the warm and wet seasons.

With the rapid development of DNA sequencing technologies, the increasing availability of genome data is enabling us to dig into bacterial genetic variability and pathogenesis. In 2002, the release of the genome sequence of ATCC 33913, the first genome sequence of *Xcc*, provided a profile of genetic information to explore the biological characteristics of *Xcc* (Ribeiro da Silva et al., 2002); however, a large repertoire of genes, including virulence factors and pathogenicity-related genes, have not been experimentally determined. Later, the genome sequence of *Xcc* 8004 became available, and comparative and functional genomic analyses provided valuable genetic information on *Xcc* pathogenicity. They found that three of unknown function genes (XC2055, XC2068 and XC2416) were located in *Xcc* 8004-specific chromosomal segments, which are responsible for the host-specific pathogenesis (Qian et al., 2005). The complete genome sequence of the *Xcc* B100 was established, where the updated annotated genome data was used to reconstruct the mechanistic model for xanthan biosynthesis (Vorhölter et al., 2008). In 2015, the draft genome sequences of strains CFBP 1869 and CFBP 5817 were determined; comparative genomics showed that core type III-secreted proteins were different between these two isolates (Bolot et al., 2015). As more genome sequences are released, we will gain further insight into interspecific genomic diversity and understanding of virulence variability between different *Xcc* isolates.

Successful infection of host tissue by bacteria often depends on the secreted bacterial virulence factors to disrupt the innate immunity. Virulence factors include bacterial toxins and adhesins that mediate bacterial attachment, cell surface-associated carbohydrates (polysaccharides and lipopolysaccharides) that protect a bacterium from hostile environments, and plant cell wall-degrading enzymes that may contribute to the pathogenicity of the bacterium (Büttner and Bonas, 2010; Li L. et al., 2019). Both EPS and LPS are surface-associated virulence factor that are major structural components of *Xanthomonas* spp. and offer protection from environmental stress (Büttner and Bonas, 2010). The type III secretion system is essential for most Gram-negative bacteria to establish infection (Puhar and Sansonetti, 2014). The core gene cluster of the *Xcc* type III secretion system consists of 25 genes and contains at least six operons (*hrpA*, *hrpB*, *hrpC*, *hrpD*, *hrpE*, and *hrpF*) (Weber et al., 2007; Li et al., 2014). *P. syringae* and *Xanthomonas*type III effectors play an important role in manipulating the host response. Pathogen effectors, like type III effectors, are major driving forces in plant pathogen co-evolution (Feng and Zhou, 2012). Pathogens may evolve new effectors or dislodge old effectors to escape host immunity. The difference in gene content, such as virulence factors, gene clusters in xanthan and LPS biosynthesis pathways, secreted proteins, and type III effectors, may contribute to genetic variability and differences in bacterial virulence.

Although, genetic diversity and population structure of the *Xcc* strains affecting cabbages in China has been revealed by MLST and Rep-PCR based genotyping (Chen et al., 2021), However, there is no detailed information on the whole genomic sequence of *Xcc* strains isolated from the main producing area of cabbage in China. Therefore, 12 *Xcc* virulent strains were isolated from seven Chinese provinces and two municipalities, and two isolates were chosen for whole-genome sequencing to compare their genetic diversity and pathogenicity. We conducted a comparative genomic analysis of BJSJQ20200612, GSXT20191014 and the known race strains including race 1 (HRIW 3811, B100, CFBP 1869), race 3 (ATCC 33913), race 4 (CFBP 5817) and race 9 (*Xcc* 8004) to understand the difference in genetic variability and virulence. Comparative genomic study clearly presented the discrepancy genes, variable genomic regions and strain specificities. The study revealed that an insight into the relationship between genetic diversity and pathogenic diversity in *Xcc*. It will lay the foundation for further study on *Xcc* virulence and provide the clue for black rot resistance breeding.

## Materials and Methods

### Bacterial Isolates and Strain Isolation

*Xcc* representative strains (HRIW 3811, HRIW 3849A, HRIW 5212, HRIW 1279A, HRIW 3880, HRIW 6181, and HRIW 8450A) were obtained from the School of Life Sciences, Wellesbourne Campus, University of Warwick, United Kingdom (HRIW). *Xcc* 8004 was obtained from Guangxi Key Laboratory of Biology for Crop Diseases and Insect Pests, Plant Protection Research Institute, Guangxi Academy of Agricultural Sciences, Nanning, China. Another 12 strains (BJTZ20191009 and BJSJQ20200612, CQ20200923, HBXT20190705 and HBCL20191115, SXXY20191115, GSXT20191014 and GSNG20191014, JSNJ20191022, GDGZ20191212, ZJHZ20191121, and SDJN20201102) were collected from cabbages from Beijing, Chongqing, Hebei, Shaanxi, Gansu, Jiangsu, Guangdong, Zhejiang and Shandong provinces in China from July 2019 to November 2020. The isolates used in this study were obtained as described by Jensen et al. (2010), with minor modifications. Briefly, diseased leaves were collected from plants with V-shape lesions, and about 1 × 1 cm-leaf tissue segments were excised from lesion margins. The leaf segments were placed into 75% ethanol for 45 s, rinsed three times in sterile distilled water, and then ground with a glass mortar and pestle with sterile distilled water, and plated on the LB medium. LB plates were monitored for the presence of characteristic convex mucoid colonies after 2 days incubation at 28°C. Single yellow-pigmented colonies were transferred to another LB agar plate for purification. The typical pale yellow colonies of *Xanthomonas* were then transferred to new LB medium and incubated for 24 h at 28°C for further purification and identification of the species. For the long-term storage, the strains were typically incubated in LB liquid media at 28°C with shaking for 24 h. And then, 500 μL of the overnight culture was added to 500 μL of 50% glycerol in a 2 mL tube, and maintained at –80°C.

### Genomic DNA Extraction From Bacterial Strains

Pure cultures were grown on LB medium at 28°C for 48 h. Genomic DNA was extracted from 2-day-old bacterial colonies grown in nutrient agar. Bacterial strains were harvested from LB culture media, and genomic DNA was extracted using the DNeasy Plant Mini Kit (TIANGEN, Beijing of China) according to the manufacture instructions. The bacterial DNA was stored at –20°C for future use.

### PCR Primers and Conditions

Sequence Characterized Amplified Region (SCAR) markers Xcc_48F/R and Xcc_53F/R were developed to amplify putative prolyl aminopeptidase and putative exported protein through whole-genome alignment (Rubel et al., 2019a). These primers were used for rapidly and accurately distinguishing *Xcc* from other pathovars. The two PCR assays were repeated independently for each strain. Primer names, sequences, and the size of the targeted product are listed in Supplementary Table 1. In addition to the 12 isolated strains, one known *Xcc* strain HRIW 3811 from cabbage and the *Pseudomonas syringae* pv. *tomato* DC3000 strain were used as the positive and negative controls, respectively. The PCR amplification was carried out using genomic DNA (gDNA) samples extracted from strains. PCR reaction volume for these primers was 10 μl including 5.0 μl of 1x Taq MasterMix (Biomed, China), 1 μl of each forward and reverse primer (10 pmol μl^–1^), 1 μl of bacterial DNA, and 2 μl of deionized water. PCR amplification conditions were standardized in a thermocycler with initial denaturation at 95°C for 5 min followed by 20 or 25 cycles of amplification with denaturation at 95°C for 30 s, annealing at different temperatures for 40 s, and elongation at 72°C for 5 min (Supplementary Table 1). A 5 μl aliquot of each amplified PCR product was loaded onto 1.5% agarose gels containing 1x TAE buffer, electrophoresed at 120 V for 30 min and checked for its respective size under UV transillumination. Confirmation of results was performed through repeated PCR amplifications. A DM2000 DNA marker (TransGen Biotech, China) was used to estimate the size of the PCR products.

### Multilocus Sequence Typing Analysis and Phylogenetic Analysis

Four housekeeping genes were chosen for MLST analysis based on previous studies (Fargier et al., 2011; Chen et al., 2021; Laala et al., 2021), including *atpD* (ATP synthase beta chain), *gyrB* (DNA gyrase subunit B), *rpoD* (RNA polymerase sigma-70 factor), and *fyuA* (TonB-dependent receptor). All PCR reaction volume for these primers was 50 μl including 25.0 μl of 2 × Primer STAR (Takara, China), 1 μl of each forward and reverse primer (10 pmol μl^–1^), 1 μl of bacterial DNA, and 22 μl of deionized water. Amplified products of these four housekeeping genes from our 12 isolated strains were purified and sequenced by the Beijing Genomics Institute (BGI, Beijing, China). The nucleotide and haplotype diversity analysis were performed using DNA Sequence polymorphism (DnaSP) software version 5.10 (Librado and Rozas, 2009). The phylogenetic tree was constructed with the four housekeeping gene concatenated sequences using Mega software version 6.06 based on the Maximum likelihood (ML) method (Tamura et al., 2013).

### Genome Sequencing and Assembly

Whole-genome sequencing of BJSJQ20200612 and GSXT20191014 were performed by the Oxford Nanopore Technologies (ONT) method in Biomarker Technologies (Beijing, China) (Deamer et al., 2016; Jain et al., 2016; Magi et al., 2018). A DNA library with 19 and 20 kb inserts were constructed, respectively. The library sequencing was quantified by PromethION 48, and filtered subreads were assembled by CANU software (version 1.5) with default parameters (Koren et al., 2017). The error-corrected assembly was tested for possible circularity using Circlator software (version 1.5.5) (Hunt et al., 2015). The genomic circle map was constructed using Circos software (version 0.66) (Krzywinski et al., 2009).

### Gene Prediction and Annotation

Protein coding sequences (CDS) were predicted using the software Prodigal v2.6.3 (Hyatt et al., 2010). tRNA and rRNA genes were identified using tRNAscan-SE v2.0 (Chan and Lowe, 2019) and Infernal v1.1.3 (Nawrocki and Eddy, 2013), respectively. The functions of the predicted proteins were annotated based on a BLASTP search against universal databases, such as the Cluster of Orthologous Groups of proteins database (COG),^1^ and the Kyoto Encyclopedia of Genes and Genomes database (KEGG),^2^ and the exclusive database: virulence factor database (VFDB)^3^ (Chen et al., 2005). Putative signal peptides and transmembrane helices were predicted using SignalP v4.0 (Petersen et al., 2011) and TMHMM v2.0 (Krogh et al., 2001), respectively.

### Genomic Comparison and Core-Genome-Based Phylogenetic Analysis of Isolated *Xanthomonas campestris* pv. *campestris* Strains

In order to design primers for specific detection of BJSJQ20200612 and GSXT20191014 isolates, we considered HRIW 3811 as a reference genome for designing primers by comparative whole-genome analyses of B100, CFBP 1869, ATCC 33913, CFBP 5817 and *Xcc* 8004, BJSJQ20200612 and GSXT20191014. The GenBank accession numbers are NZ_CP025750 for HRIW 3811, AM920689 for B100, NZ_CM002545 for CFBP 1869, AE008922 for ATCC 33913, NZ_CM002673 for CFBP 5817, NC_007086 for *Xcc* 8004, CP069085 for BJSJQ20200612, and CP069084 for GSXT20191014, respectively. The homology between sequence blocks among *Xcc* strains (shown by differential colors) was performed using mauve software (version 2.4.0). We developed specific primers to classify isolates from differential IS regions. The whole predicted protein sequences from BJSJQ20200612, GSXT20191014 and 33 completely sequenced or draft *Xcc* genomes were used to construct the phylogenetic tree. The core-genome was extracted from the whole genomes of *Xcc* strains using the USEARCH program (ver. 9.0). The concatenated amino acid sequences of the core-genome were aligned using MUSCLE v3.8.31 (Edgar, 2004). A core-genome-based phylogenetic tree was constructed from the concatenated sequences using the phyML version 3.0 by the maximum likelihood method (Guindon et al., 2005).

### The Development of Specific Molecular Markers for BJSJQ20200612 and GSXT20191014

A genome-wide alignment among the sequences of B100, HRIW 3811, CFBP 1869, ATCC 33913, CFBP 5817, *Xcc* 8004, BJSJQ20200612, and GSXT20191014 was performed with Mauve multiple genome alignment tools. We identified several DNA fragments by comparative sequence alignments that were unique to either *Xcc* strain BJSJQ20200612 or *Xcc* strain GSXT20191014. Highly conserved regions were excluded when primers were designed. The variable genomic regions (> 800 bp) in the genomic sequence of BJSJQ20200612 and GSXT20191014 isolates compared to published strains were chosen to design the specific markers. Primers XccSJQ-12, XccSJQ-30, XccSJQ-31, XccSJQ-62 and XccXTZ-10, XccXTZ-16, XccXTZ-24, XccXTZ-28 were designed to detect BJSJQ20200612 and GSXT20191014 isolates, respectively (Supplementary Table 2). Among these designed primers, XccSJQ-12, XccSJQ-30, XccSJQ-31 and XccSJQ-62 anchored in the BJSJQ20200612 genomic sequence from 1,221,404 to 1,222,047 bp, 3,077,954–3,078,605 bp, 3,106,515–3,107,131 bp, 620,025–621,536 bp, respectively. XccXTZ-10, XccXTZ-16, XccXTZ-24 and XccXTZ-28 located at the position from 101,571 to 102,074 bp, 166,619–167,210 bp, 2,469,077–2,469,769 bp, 2,866,934–2,867,550 bp in GSXT20191014 genome, respectively. The PCR reaction mixture (10 μl) included 1 μl of gDNA, 10 pmol of each forward and reverse primer, and 7 μl of enzyme mixture. The conditions for PCR amplification for marker XccSJQ-62 was 95°C for 5 min followed by 25 cycles (95°C for 30 s, 67°C for 30 s, and 72°C for 60 s) and a final extension at 72°C for 10 min. The annealing temperature of XccSJQ-12, XccSJQ-30 and XccSJQ-31 was decreased to 65°C for 30 s. The annealing temperature of XccXTZ-10, XccXTZ-16, XccXTZ-24, and XccXTZ-28 was increased to 70°C for 30 s. The amplified PCR products were detected using gel electrophoresis (1.5% agarose gel).

### Pathogenicity Testing by Artificial Inoculation of the Purified *Xanthomonas campestris* pv. *campestris* Strains

Twelve isolated strains were finally selected for subsequent studies. For all biological assays, the strain HRIW 3811 was used as the positive control, and sterile water was used as the negative control. Strains preserved at –80°C were defrosted and grown in LB medium (5 ml) at 28°C for 24 h. The bacterial suspension was centrifuged for 5 min at 5,000 g, and the supernatant was discarded; then, the bacterial concentration was adjusted to 1 × 10^8^CFU ml^–1^. Cabbage inbred line 1371 seeds were sown in plastic pots with sterilized substrate (peat: vermiculite = 2:1) and cultivated in a greenhouse at 24 ± 2°C with 16/8 h (light/dark). The seedlings with four fully expanded leaves were used for pathogen inoculation. For pathogenicity assays, the two youngest leaves were inoculated by dipping clipped secondary veins (with sterile scissors) into purified *Xcc* strain suspensions (1 × 10^8^ CFU ml^–1^) (Vicente et al., 2001; Gu et al., 2008; Afrin et al., 2018a). Ten plants were inoculated per isolate; reference *Xcc* strain HRIW 3811 and water were used as the positive and negative controls. Inoculated plants were grown in a growth chamber at 24 ± 2°C with 16/8 h (light/dark) and 70–75% relative humidity. Plants were recorded for typical black rot symptoms with progressive V-shaped and yellow necrotic lesions with blackened veins after 5–14 days. The average lesion area/health area ratio from 20 leaves was measured with Adobe Photoshop 7.0.1. The method of pathogenicity testing on other brassica crops was same as above.

## Results

### Identification of Bacterial Strains

**FIGURE 1:**
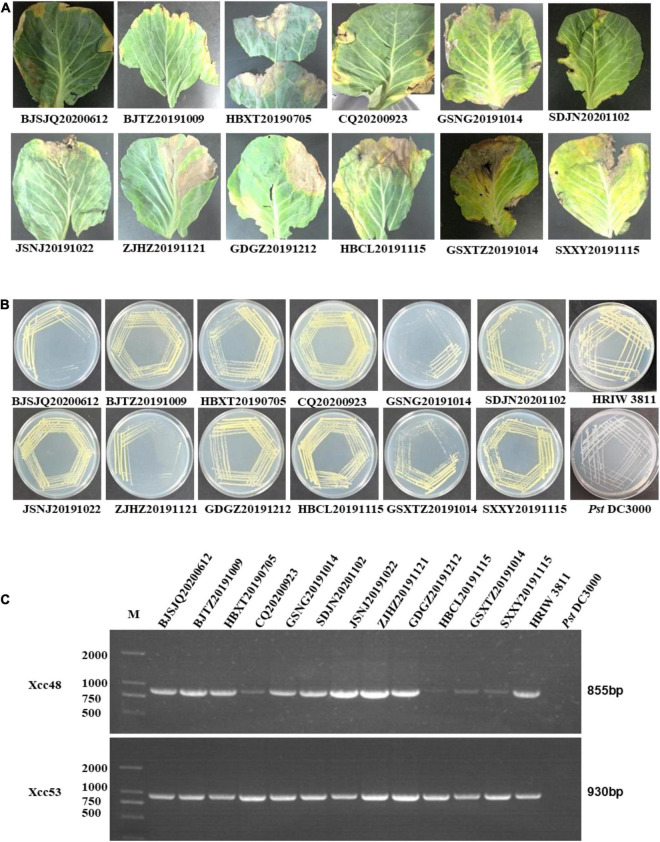
Purified colony morphology isolated from diseased cabbage leaf samples and specific molecular detection of *Xcc* in cabbage. **(A)** Diseased leaves collected in different cabbage growing regions of China. **(B)** Purified strain colonies growing on LB medium. **(C)** Detection of the isolated strains by *Xcc*-specific markers, where Xcc_48F/R and Xcc_53F/R were used to distinguish *Xcc* strains from other bacterial species. Isolated strains: BJTZ20191009 and BJSJQ20200612, CQ20200923, HBXT20190705 and HBCL20191115, SXXY20191115, GSXT20191014 and GSNG20191014, JSNJ20191022, GDGZ20191212, ZJHZ20191121 and SDJN20201102 were collected from Beijing, Chongqing, Hebei, Shaanxi, Gansu, Jiangsu, Guangdong, Zhejiang, and Shandong provinces in China. HRIW 3811 and *Pseudomonas syringae* pv. *tomato* DC3000 were used as the positive and negative control, respectively.

Twelve bacterial isolates were obtained from black rot diseased leaves (with V-shape lesions), which were harvested from cabbage-growing regions in Beijing, Chongqing, Hebei, Shaanxi, Gansu, Jiangsu, Guangdong, Zhejiang, and Shandong provinces in China from July 2019 to November 2020 (Figure 1A). Twelve of the purified bacterial isolates exhibited the typical yellow mucoid and glistening colonies and are suggestive of *Xcc* bacteria (Figure 1B). Xcc_48F/R and Xcc_53F/R were used to determine whether the isolates were the *Xcc* or other pathovars. Positive PCR amplification of 855 and 930 bp were observed in the 12 isolates and *Xcc* control (HRIW 3811), respectively (Figure 1C). The colonial morphology and PCR results indicated that the 12 isolates are *Xcc* pathovar.

### Pathogenicity Analysis

**FIGURE 2:**
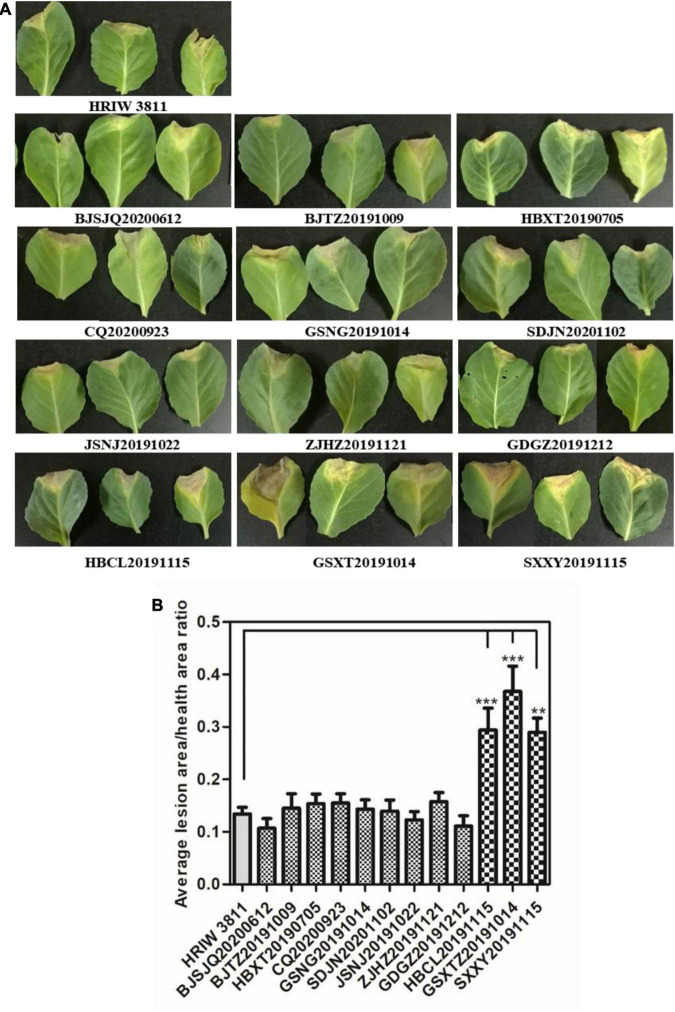
Pathogenicity analysis of isolated strains on cabbage. **(A)** All 12 isolated *Xcc* strains caused typical V-shaped lesions in the cabbage after leaf clipping inoculation. The average lesion area/health area ratio (per leaf) caused by three isolated strains (HBCL20191115, SXXY20191115, and GSXT20191014) were larger than that caused by the positive control (HRIW 3811 strain). Photos were taken at 7 d post-inoculation. **(B)** The average lesion area/health area ratio triggered by the isolated strains were bigger than that caused by HRIW 3811. Lesion and health area were measured using 20 leaves samples at 10 d post-inoculation (data are means ± SD; *n* > 3; **P < 0.01 and ***P < 0.001).

All 12 isolated strains were evaluated for their pathogenicity in cabbage by the leaf-clipping inoculation method. Seedlings inoculated with sterile water and HRIW 3811 were used as the negative and positive controls, respectively. The distinct V-shaped lesions were observed in all inoculated cabbages after 14 days (Figure 2A). Additionally, we also found that the isolated strains HBCL20191115, SXXY20191115 and GSXT20191014 induced more severe symptoms in cabbage inbred line 1371 than HRIW 3811, and the average lesion area/health area ratio (per leaf) caused by these three isolated strains were larger than that caused by the positive control (HRIW 3811 strain) (Figure 2B). However, the results showed that there was no difference in pathogenicity between the rest of the strains and HRIW 3811. Taken together, these results show that HBCL20191115, SXXY20191115 and GSXT20191014 (from the field) were more virulent than HRIW 3811 in cabbage inbred line 1371. Meanwhile, the virulence experiments were also tested in the Chinese cabbage, radish and broccoli; the pathogenicity results also showed that the isolate GSXT20191014 was more aggressive than BJSJQ20200612 and HRIW 3811 on these three brassica crops (Supplementary Figure 1).

### Phylogenetic Analysis of Housekeeping Genes

**FIGURE 3:**
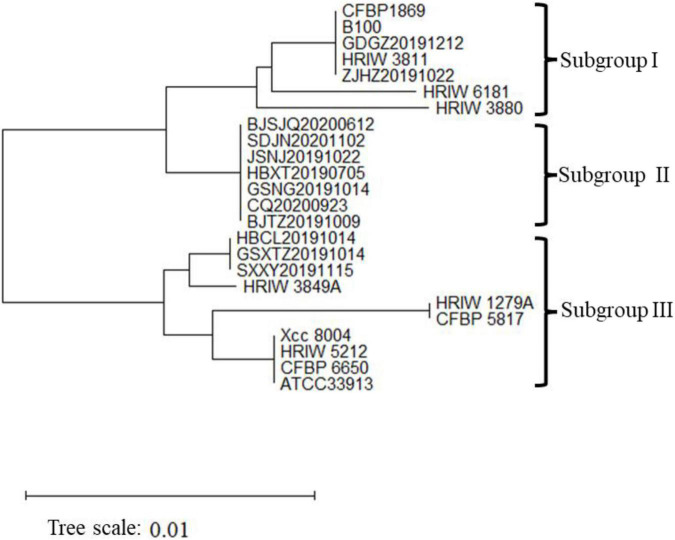
Neighbor-joining tree constructed with concatenated partial sequences of four housekeeping gene (*atpD*, *gyrB*, *rpoD*, and *fyuA*) of our 12 isolated strains and 12 different *Xcc* race strains. The tree is drawn to scale, with branch lengths in the same units as those of the evolutionary distances used to infer the phylogenetic tree. The tree scale represents 0.01.

The length of these four housekeeping gene fragments (*atpD*, *gyrB*, *rpoD*, and *fyuA*) were 648, 705, 807, and 771 bp, and the total length of four housekeeping gene concatenated sequences was 2,931 bp. The number of polymorphic nucleotide sites among the strains varied from 13 to 107 (Supplementary Table 3). Phylogenetic analysis was performed by the concatenated sequences of four individual genes of 24 *Xcc* strains. Our isolated *Xcc* samples were distributed in three subgroups in the phylogenetic relationship (Figure 3). GDGZ20191212 and ZJHZ20191121 were distributed into subgroup I, which also consist of three race1 isolates CFBP1869, HRIW3811 and B100. Seven strains including BJSJQ20200612, BJTZ20191009, HBXT20190705, CQ20200923, GSNG20191014, SDJN20201102, and JSNJ20191022 were divided into the largest subgroup II, which has probably evolved from race 1. Interestingly, HBCL20191115, HBXT20190705 and GSXT20191014 were very closely related to race 2 (HRIW3849A), which were classified into subgroup III. Meanwhile, race 3 (ATCC33913), race 4 (CFBP5817), and race 9 (*Xcc* 8004) were divided into the same subgroup but in different clades. Taken together, our isolated *Xcc* strains were classified into three different genetic subgroups.

### General Genomic Features of BJSJQ20200612 and GSXT20191014

**FIGURE 4:**
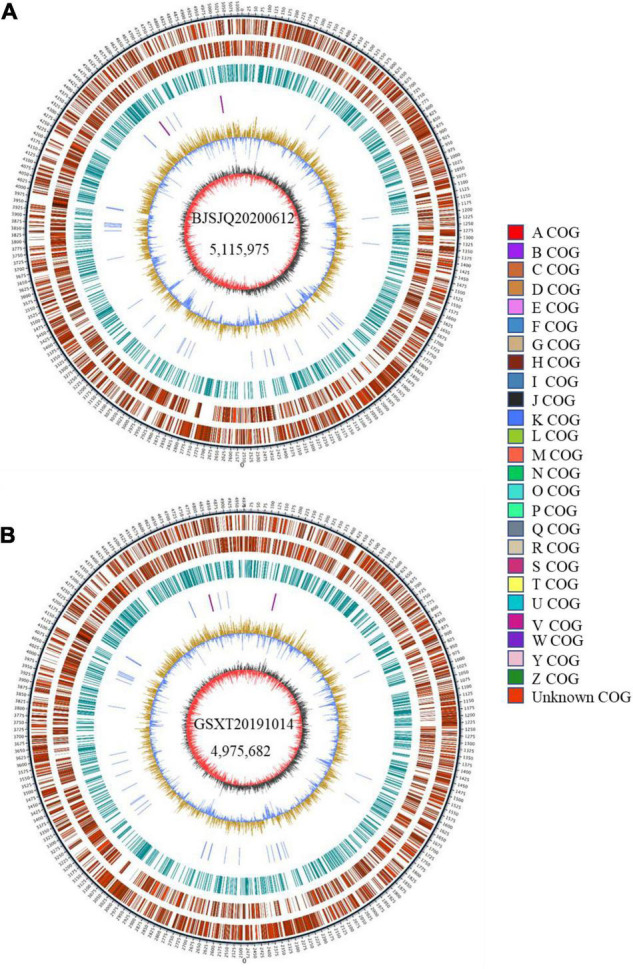
The Graphical circular map of the *Xcc* isolates BJSJQ20200612 and GSXT20191014. **(A)** Circular map of BJSJQ20200612. The genome of BJSJQ20200612 is composed of a single circular chromosome that is 5,115,975 bp in size with no apparent autonomous plasmids. **(B)** Circular map of GSXT20191014. The final assembly of the GSXT20191014 genome resides on a single circular chromosome with a size of 4,975,682 bp. Circles range from 1 to 7 (outer circle to inner circle). Circle 1: The size and position of the whole genome sequence, each scale represent 5 kb; circles 2 and 3: Genes on the positive and negative chains of the genome, respectively, and different colors represent different COG functional classification; circle 4: repeating sequence; circle 5: tRNA and rRNA genes; circle 6: GC content, the light yellow part indicates that the GC content in this region is higher than the average GC content in the genome, and the higher the peak value, the greater the difference between the region and the average GC content. The blue part indicates that the GC content in this region is lower than the average GC content in the genome; Circle 7: Is GC-skew, where dark gray represents the area where G content is greater than C, and red represents the area where C content is greater than G.

There were 43,522 and 35,039 raw sequences obtained from BJSJQ20200612 and GSXT20191014 isolates, respectively. We obtained 39,675 and 32,258 clean sequences after filtering out adapters and low quality and short segment (length < 2,000 bp) reads, respectively. Furthermore, we got the 158x and 140x genome coverages for isolates BJSJQ20200612 and GSXT20191014, respectively. The completeness of the genome sequences of BJSJQ20200612 and GSXT20191014 isolates is 99.67 and 99.55%, and there is no contamination in both isolates. The final assembly of the BJSJQ20200612 genome resides on a single circular chromosome with a size of 5,115,975 bp (Figure 4A). The genome of GSXT20191014 is composed of a single circular chromosome that is 4,975,682 bp in size without apparent autonomous plasmids (Figure 4B). The average G + C content of the genome is 64.99 and 65.29% of the BJSJQ20200612 and GSXT20191014 genomes, respectively, which is similar to HRIW 3811 (65.1%), B100 (65%), CFBP 1869 (65%), ATCC 33913 (65.1%), CFBP 5817 (65.2%), and *Xcc* 8004 (64.94%) (Table 1). The genome of GSXT20191014 possesses the highest G + C content in these sequenced *Xcc* strains. There were 4,382 and 4,184 predicted protein-coding genes in the genome of BJSJQ20200612 and GSXT20191014, respectively. Additionally, the BJSJQ20200612 genomic sequences had 181 RNA genes, including 56 tRNA genes, six rRNA genes (16S rRNA, 5S rRNA, and 23S rRNA), and 119 ncRNAs; whereas, the GSXT20191014 chromosome contained six rRNA genes, 57 tRNA genes, and 120 ncRNA genes (Table 1). The total repetitive sequence length is 49,353 bp and 61,786 bp in BJSJQ20200612 and GSXT20191014 isolates, accounting for 0.96 and 1.24% of the whole genome sequences, respectively. Functional categorization of the coding DNA sequences (CDSs) in BJSJQ20200612 and GSXT20191014 were analyzed using COG database. The results indicated that 32.25 and 33.14% predicted genes are related to metabolism, 23.06 and 23.49% of predicted genes are correlated with cellular processes and signaling, 16.76 and 16.87% are connected with information storage and processing. Furthermore, 27.95 and 26.51% of genes are poorly characterized in BJSJQ20200612 and GSXT20191014 genomes, respectively (Supplementary Table 4).

**TABLE 1:** Genome features of BJSJQ20200612 and GSXT20191014 and other sequenced *Xcc* strains.

Strains	Accession	Genome size (bp)	G + C Content (%)	Total genes	Predicted no. of CDS	Ribosomal RNA (no.)	Transfer RNA(no.)	Other RNA(no.)	Pseudogene (no.)
HRIW 3811 (United States)	No_CP025750	5,072,566	65.1	4,661	4,661	6	53	90	75
B100 (United Kingdom)	AM920659	5,079,002	65.0	4,576	4,471	2	54	89	119
CFBP 1869 (Côte d’Ivoire)	NZ_CM002545	5,011,687	65.0	4,535	4,535	3	53	90	131
ATCC 33913 (United Kingdom)	AE008922	5,076,187	65.1	4,607	4,181	2	54	93	114
CFBP 5817 (Chile)	NZ_CM002673	4,918,955	65.2	4,432	4,432	3	53	92	132
*Xcc* 8004 (United Kingdom)	NC_007086	5,148,708	64.94	4,371	4,273	2	54	93	119
BJSJQ20200612 (China)	CP069085	5,115,975	64.99	4,382	4,382	6	56	119	2
GSXT20191014 (China)	CP069084	4,975,682	65.29	4,184	4,184	6	57	120	1

*No. denotes that the predicted number of Ribosomal RNA, Transfer RNA, other RNA, and Pseudogene in different genomes, respectively.*

### Relatedness of *Xanthomonas campestris* pv. *campestris* Strains Based on Core-Genomes

**FIGURE 5:**
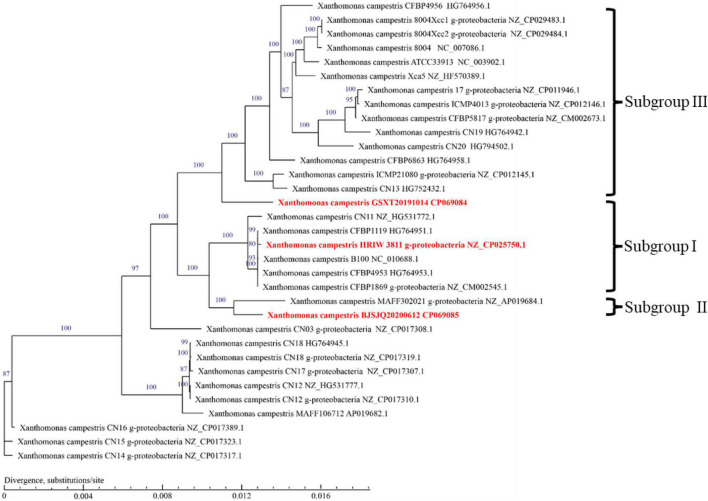
A phylogenetic tree with bootstrap values (1,000 bootstrap replications) reconstructed using the concatenated amino acid sequences of *Xcc* core-genome showing the relationships among *Xcc* strains. Strain names as described in GenBank are used in the tree and the type strains are highlighted in red bold. The bar indicates divergence, substitutions per site.

To infer the phylogenetic relationships among *Xcc* strains, a phylogenetic tree was constructed using the concatenated amino acid sequences of genes in the core-genome (Figure 5). Unlike the phylogenetic tree based on housekeeping gene sequences, the phylogenetic tree based on the core-genome showed that *Xcc* strains have been more clearly separated into different phylogenetic lineages. It is generally accepted that closely related organisms share more orthologous genes, suggesting that evolutionary relationships among *Xcc* can be inferred by the presence/absence of orthologous genes. The results showed that BJSJQ20200612 was very closely related to isolate MAFF302021, isolated from Shiga, Japan. Interestingly, these two pathogens form a clade; these pathogens may have evolved from the same ancestor. Additionally, BJSJQ20200612 and MAFF302021 isolates shared a close relationship with HRIW 3811, B100 and CFBP 1869 sequences, which were commonly recognized as *Xcc* race 1. GSXT20191014 has the highest number of unique genes in this subgroup, formed a phylogenetic lineage clearly distinct from race 3 (ATCC33913), race 4 (CFBP5817), and race 9 (*Xcc* 8004) strains, which may reflect a different characters of GSXT20191014 from those of other *Xcc.* Interestingly, BJSJQ20200612 and GSXT20191014 isolates were also classified into two subgroups of *Xcc* according to the core-genome-based phylogenetic tree, which was consistent with subgrouping data performing by the MLST and specific molecular markers experiments.

### Validation of the Developed Specific Molecular Markers for BJSJQ20200612 and GSXT20191014 Isolates

**FIGURE 6:**
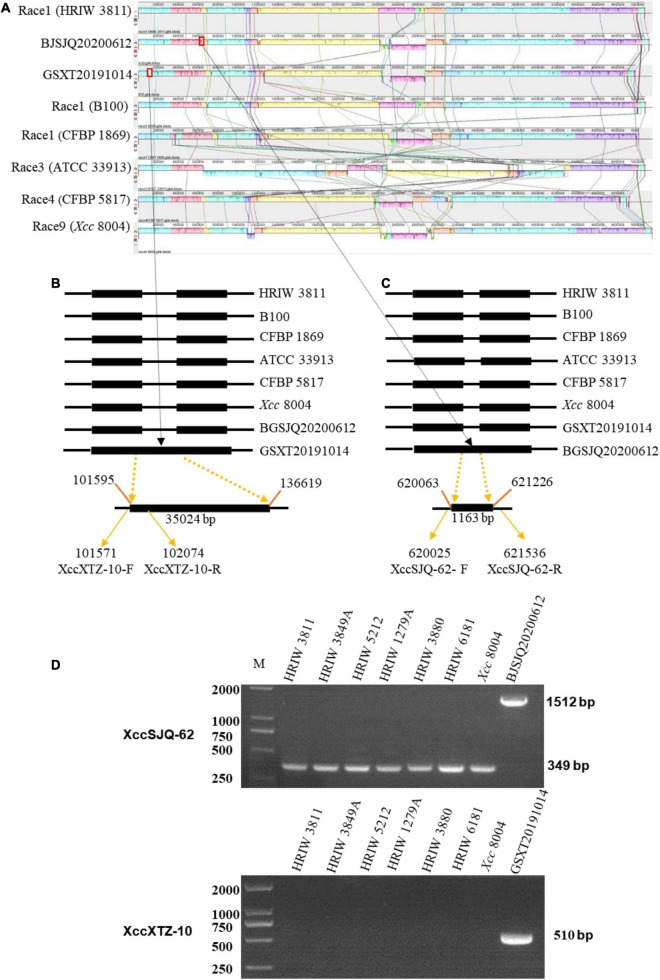
Multiple alignments of the whole genome sequence of *Xcc* strains by Mauve (version 2.4.0) and the development of molecular markers. **(A)** Alignment of the whole sequence of eight *Xcc* strains (B100, HRIW 3811, CFBP 1869, ATCC 33913, CFBP 5817, and *Xcc* 8004). HRIW 3811 was used as the reference genome. Each genome is laid out horizontally with homologous segments outlined as colored rectangular solid boxes. The syntenic blocks are connected among the strains with lines. The rectangular boxes (red outline) indicate the positions of inserted sequence in the genome. **(B)** An insertion sequence of BJSJQ20200612 isolate relative to other *Xcc* strains are set with the line diagram. A 35,024 bp large insertion was located from 101,595 to 136,619 of the genomic sequence. XccXTZ-10-F/R were the GSXT20191014-specific molecular markers. **(C)** An IS5 family transposase insertion in the GSXT20191014 isolate compared to other *Xcc* strains are shown with the line diagram. XccSJQ-62-F/R were the BJSJQ20200612-specific molecular marker. The GenBank accession numbers are NZ_CP025750 for HRIW 3811, AM920689 for B100, NZ_CM002545 for CFBP 1869, AE008922 for ATCC 33913, NZ_CM002673 for CFBP5817 and NC_007086 for *Xcc* 8004, respectively. **(D)** The usability of molecular markers XccSJQ-62 and XccXTZ-10 were tested by PCR using the genomic DNA of *Xcc* strains (HRIW 3811, HRIW 3849A, HRIW 5212, HRIW 1279A, HRIW 3880, HRIW 6181, and *Xcc* 8004). DNA concentration of all samples was 50 ng/μl. Lane M: 2k plus DNA ladder was used as the size marker. BJSJQ20200612 and GSXT20191014 were used as the positive control on the upper and lower panel, respectively.

A genome-wide alignment among the sequences of BJSJQ20200612, GSXT20191014 and other sequenced *Xcc* strains was performed with Mauve multiple genome alignment tools (Figure 6A). XccSJQ-62 and XccXTZ-10 were chosen as the representative examples for the development of specific molecular markers for BJSJQ20200612 and GSXT20191014 isolates. An indel marker (XccXTZ-10) was designed according to a specific 35,024 bp insertion (101,595–136,619) in the genomic sequence of strain GSXT20191014 (Figure 6B). XccXTZ-10 amplified a 510 bp product in the GSXT20191014 isolate (Figure 6D). Equally, we found an 1,163 bp insertion at the BJSJQ20200612 genomic position 620,018–621,536, where a gene (*IS5*) encoding an IS5 family transposase was located and the sequence characterized amplified region primer set (SCAR; XccSJQ-62) was developed (Figure 6C). The PCR results showed a 1,512 bp product was specifically amplified in strain BJSJQ20200612, while a band size of 349 bp was observed in other strains (Figure 5D). Additionally, the PCR results showed that 644, 652, and 617 bp products were amplified in the BJSJQ20200612 genome using the XccSJQ-12, XccSJQ-30 and XccSJQ-31 markers. Meanwhile, XccXTZ-16, XccXTZ-24 and XccXTZ-28 markers amplified the 630, 693, and 617 bp products from GSXT20191014 genome, respectively (Supplementary Figure 2). A transposase mediated insertion and a large DNA fragment insertion were used to develop the molecular marker for bacteria identification. There are a variety of insertions in the genomic sequence of BJSJQ20200612 and GSXT20191014 isolates. Above-mentioned PCR data are consistent with the expected results according to our comparative comparison of B100, HRIW 3811, CFBP 1869, ATCC 33913, CFBP 5817, *Xcc* 8004, BJSJQ20200612, and GSXT20191014 genomes. Taken together, the developed markers (XccSJQ-12, XccSJQ-30, XccSJQ-31, XccSJQ-62 and XccXTZ-10, XccXTZ-16, XccXTZ-24, XccXTZ-28) could be used for distinguishing BJSJQ20200612 and GSXT20191014 isolates, respectively.

### Subgrouping of Unknown Isolated *Xanthomonas campestris* pv. *campestris* Strains by the Specific Molecular Markers

**FIGURE 7:**
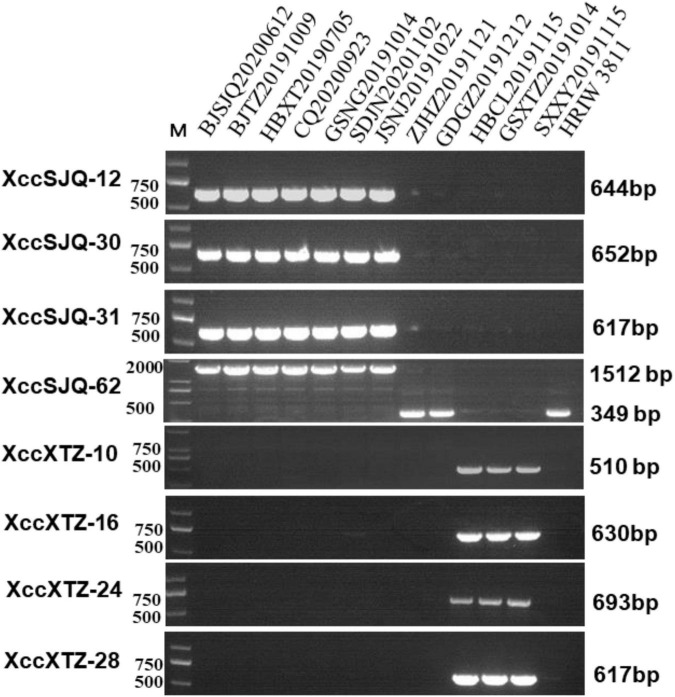
Subgrouping of the unknown *Xcc* strains by the specific molecular markers. The isolated strains were tested by specific molecular markers XccSJQ-12, XccSJQ-30, XccSJQ-31, XccSJQ-62, XccXTZ-10, XccXTZ-16, XccXTZ-24, and XccXTZ-28 for strain classification. DNA concentration of all samples was 50 ng/μl. Lane M: 2k plus DNA ladder was used as a size marker. Lane1–13: *Xcc* strains (BJSJQ20200612 and BJTZ20191009, HBXT20190705, CQ20200923, GSNG20191014, SDJN20201102, JSNJ20191022, ZJHZ20191121, GDGZ20191212, HBCL20191115, GSXT20191014, and SXXY20191115, and HRIW 3811, respectively).

Isolate-specific markers XccSJQ-12, XccSJQ-30, XccSJQ-31, XccSJQ-62 and XccXTZ-10, XccXTZ-16, XccXTZ-24, XccXTZ-28 were also used to determine other isolated strains. The PCR results indicated that the XccSJQ-12, XccSJQ-30, XccSJQ-31 and XccSJQ-62 primer sets amplified the 644, 652, 617, and 1,512 bp bands from the BJSJQ20200612 isolate and the isolates: BJTZ20191009, HBXT20190705, CQ20200923, GSNG20191014, SDJN20201102, and JSNJ20191022. However, there were no bands produced by markers (XccSJQ-12, XccSJQ-30 and XccSJQ-31) and a 349 bp product generated by marker XccSJQ-62 from the HBCL20191115, SXXY20191115, GSXT20191014, ZJHZ20191121, and GDGZ20191212 isolates. Likewise, a 510 bp band were specifically amplified by marker XccXTZ-10 and no band was observed by the primer sets XccXTZ-16, XccXTZ-24 and XccXTZ-28 from the GSXT20191014, HBCL20191115 and SXXY20191115 isolates (Figure 7). Taken together, we concluded that our isolated strains could be categorized into three subgroups according to eight isolate-specific markers: isolates ZJHZ20191121 and GDGZ20191212 belong to race 1 (HRIW 3811-like isolates); BJSJQ20200612, BJTZ20191009, HBXT20190705, CQ20200923, GSNG20191014, SDJN20210102, and JSNJ20191022 are classified as subgroup II, and isolates HBCL20191115, SXXY20191115 and GSXT20191014 are considered subgroup III.

### The Variable Genomic Regions in the BJSJQ20200612 and GSXT20191014 Genomes

In order to analyze the genomic variability of our isolated *Xcc* bacteria, we performed comparative genomic alignment analysis of BJSJQ20200612 and GSXT20191014 with five available complete genomes of *Xcc* isolates HRIW 3811, B100, CFBP 1869, ATCC 33913, CFBP 5817, and *Xcc* 8004 by Mauve software (version 2.4.0). We found 26 variable genomic regions (> 800 bp) in the genomic sequence of BJSJQ20200612 isolate compared to these five published strains. The length of variable genomic regions ranged from 884 bp to 31,905 bp. It should be noted that seven variable genomic regions larger than 5,000 bp, which are located at positions: 255,690–260,986, 1,199,954–1,223,227, 1,214,274–1,223,224, 2,500,995–2,510,117, 3,048,482–3,056,141, 3,074,557–3,080,689, and 3,086,987–3,118,892 bp of the genome sequence (Supplementary Table 5). In addition, 47 variable genomic regions (> 1,000 bp) were found in the genomic sequence of GSXT20191014; the size of the variable genomic regions ranged from 1,077 to 49,749 bp. There were five variable genomic regions larger than 5,000 bp, which are located at positions: 101,594–136,624, 1,244,066–1,252,479, 1,253,110–1,259,066, 2,450,290–2,500,038, and 2,505,237–2,520,734 bp (Supplementary Table 5). The variable genomic regions are rich in transposable elements. The IS3, IS4 and IS5 families are highly represented in both isolates (Supplementary Table 5). Most of these ISs are located near isolate-specific genes, which usually are associated with bacterial genome rearrangements.

### Virulence Factors in the BJSJQ20200612 and GSXT20191014 Genomes

A total of 820/830 known or putatively related genes encoding virulence factors were detected in the BJSJQ20200612 and GSXT20191014 genome, respectively (Supplementary Tables 6, 7). Furthermore, there were six and ten virulence factors identified inside the variable genomic regions of BJSJQ20200612 and GSXT20191014 genomes, respectively. These virulence factors, including toxin and surface-associated carbohydrates, such as zona occludens toxin, methyl-accepting chemotaxis protein (encoding by *cheD* gene), type IV secretion system component VirD4, were found in both isolates. Interestingly, the gene GE002595 (*fhaB*) encoding filamentous hemagglutinin (FHA) proteins were uniquely identified at the variable genomic regions in the GSXT20191014 genome. GE002595 has function in the two-partner secretion pathway (TPS), which is responsible for bacterial adherence. Meanwhile, it was important to point out that *virB1* (GE000326) encoding type IV secretion system attachment mediating protein was also found at the variable genomic regions of the GSXT20191014 genome (Table 2).

**TABLE 2:** The virulence factors in the variable genomic regions of BJSJQ20200612 and GSXT20191014 genomes according to the VFDB database.

	Site	Position	Gene ID	Gene name in VFDB	Gene function in VFDB
BJSJQ20200612	1	255,690–260,986	GE000211	*fleS/flrB*	Two-component sensor kinase
			GE000212	*pilR*	Two-component response regulator PilR
	5	826,174–827,374	GE000695	*virD4*	Type IV secretion system component VirD4
	14	2,500,995–2,510,117	GE002116	*cheD*	Methyl-accepting chemotaxis protein
	15	2,526,584–2,529,582	GE002137	*cheD*	Methyl-accepting chemotaxis protein
	23	3,086,987–3,118,892	GE002618	*virD4*	Type IV secretion system component VirD4
GSXT20191014	1	101,594–136,624	GE000321	*aha_1389*	CobQ/CobB/MinD/ParA family protein
			GE000326	*virB1*	Type IV secretion system attachment mediating protein VirB1 Homolog
			GE000328	*virD4*	Type IV secretion system component VirD4
	16	1,244,066–1,252,479	GE001264	*allS*	DNA-binding transcriptional activator AllS
	26	2,450,290–2,500,038	GE002271	*zot*	Zona occludens toxin
			GE002297	*aha_1389*	CobQ/CobB/MinD/ParA family protein
	27	2,505,237–2,520,734	GE002312	*oatA*	Peptidoglycan O-acetyltransferase
	31	2,760,425–2,762,064	GE002510	*cheD*	Methyl-accepting chemotaxis protein
	32	2,763,221–2,764,771	GE002512	*cheD*	Methyl-accepting chemotaxis protein
	33	2,863,691–2,868,643	GE002595	*fhaB*	Filamentous hemagglutinin/adhesin

*VFDB represents virulence factor database.*

A total of 665 and 632 proteins were predicted to be secreted in the BJSJQ20200612 and GSXT20191014 genomes by the SignalP and TMHMM software, respectively (Supplementary Table 8). Furthermore, the secreted proteins were also identified inside the variable genomic regions of BJSJQ20200612 and GSXT20191014. One lytic murein transglycosylase, one beta-propeller fold lactonase family protein and five hypothetical proteins were present in the variable genomic regions of the BJSJQ20200612 genome. Additionally, two copies of peptidase S8/S53 were found at site 1 and 26 of the variable genomic regions of the GSXT20191014 genome (Table 3). The gene GE002595 (*fhaB*) encoding FHA proteins were identified at the variable genomic regions in the GSXT20191014 genome.

**TABLE 3:** The secreted proteins in the variable genomic regions of BJSJQ20200612 and GSXT20191014 genomes.

	Site	Secreted protein	Position	Nucleotide similarity of related proteins (%)					
				ATCC 33913	*Xcc* 8004	B100	HRIW 3811	GSXT 20191014	BJSJQ 20200612
BJSJQ20200612	1	Hypothetical protein	255,690–260,986	None	None	None	None	None	GE000213
	9	Hypothetical protein	1,214,274–1,223,224	None	None	None	None	None	GE001021
	13	Lytic murein transglycosylase	2,463,905–2,466,201	None	None	None	None	None	GE002078
	20	Hypothetical protein	3,074,557–3,080,689	None	None	None	None	None	GE002604
		Hypothetical protein		None	None	None	None	None	GE002605
	23	Beat-propeller fold lactonase family protein	3,086,987–3,118,892	None	None	None	None	None	GE002634
	25	Hypothetical protein	3,291,469–3,292,933	100%	100%	100%	100%	100%	GE002800
GSXT20191014	1	S8/S53	101,594–136,624	None	None	None	None	GE000333	None
	3	TonB-dependent receptor	188,308–189,666	93.70%	93.38%	93.26%	93.38%	GE000385	93.26%
	16	Hypothetical protein	1,244,066–1,252,479	100%	100%	None	None	GE001260	None
	26	Hypothetical protein	2,450,290–2,500,038	None	None	None	None	GE002268	None
		S8/S53		None	None	None	None	GE002293	None
	33	Filamentous hemagglutinin	2,863,691–2,868,643	78.53	78.73	None	None	GE002595	None
	45	Ankyrin repeat domain-containing protein	4,706,689–4,707,766	76.11%	75.99%	None	None	GE00034	76.92%
	46	TonB-dependent receptor	4,825,920–4,829,868	None	99.70%	99.08%	99.08%	GE000121	99.04%

*None represents that there is no related protein in this genome.*

**FIGURE 8:**
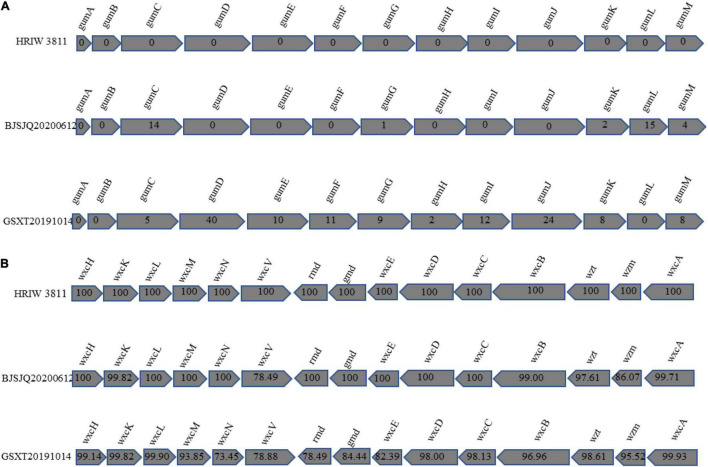
Comparative representation of the organization of *Xcc* gene clusters directing the synthesis of xanthan and lipopolysaccharide. The *Xcc* genes involved in the synthesis of xanthan are symbolized by shaded arrows that illustrate the similarities in size, order, and direction of transcription among these genes. Conventional gene names are given above the arrows of homologous genes. **(A)** Genes *gumB* to *gumM* are required for xanthan synthesis. The numbers within the arrows provide the numbers of the nucleotides deviating by SNPs or insertion-deletion events from the BJSJQ20200612 and GSXT20191014 gene sequences compared to the HRIW 3811. **(B)** The genes *wxcH*, *wxcL*, *wxcM*, *wxcN*, *wxcO*, *rmd*, *gmd*, *wxcE*, *wxcD*, and *wxcC* are required for lipopolysaccharide synthesis. The numbers within the arrows show the percentage of nucleotide similarity among BJSJQ20200612, GSXT20191014 and HRIW 3811.

In *Xanthomonas* bacteria, the biosynthesis of EPS is regulated by the highly conserved *gum* gene cluster, which comprises 12 genes from *gumB* to *gumM* (Becker et al., 1998). Our results showed that there were few single-nucleotide polymorphisms (SNPs) distinguishing HRIW 3811 and BJSJQ20200612 isolates in these 12 gene sequences. However, more differences in these gene sequences were observed between HRIW 3811 and GSXT20191014 isolates (Figure 8A). LPS production is directly controlled by the *wxc* gene cluster, which consists of 15 genes (Vorholter et al., 2001). They were conserved between HRIW 3811 and BJSJQ20200612 isolates, only *wxcV* and *wxzm* genes showed low similarity, but 10 genes (*wxcH*, *wxcL*, *wxcM*, *wxcN*, *wxcO*, *rmd*, *gmd*, *wxcE wxcD*, and *wxcC*) were identical. However, five genes (*wxcN*, *wxcV*, *rmd*, *gmd*, and *wxcE*) in the GSXT20191014 genomic sequence showed less than 85% similarity compared to those in the HRIW 3811 isolate (Figure 8B). However, the differences in production of EPS or LPS need be further studied.

The type III secretion system plays an important role in bacterial pathogenicity. BJSJQ20200612 and GSXT20191014 genomes contain a highly conserved type III gene cluster (including *hrp* and *hrc*) compared to that in the HRIW 3811 genome, covering 23,112 and 23,115 kb with 25 open reading frames (ORFs), respectively (Supplementary Table 9). However, secreted effectors via type III are different in these two genomes. The type III effectors were predicted and identified using the *Xanthomonas* resource.^4^ ATCC 33913 and *Xcc* 8004 contain 33 type III effector genes. Twenty-seven effector genes were present in the BJSJQ20200612 genome; and 23 effector genes were identified in GSXT20191014 genome. We identified 18 common effectors, these effector genes (*avrBs2*, *xopN*, *xopQ*, *xopAY*, *xopAM*, *xopF*, *hpaW*, *hpaA*, *xopA*, *xopAL*, *xopP*, *xopZ*, *avrXccA2*, *xopAC*, *xopK*, *xopAG*, *xopL*, and *avrXccA1*) are present in all six sequenced *Xcc* genomes (ATCC 33913, *Xcc* 8004, B100, HRIW 3811, BJSJQ20200612, and GSXT20191014) (Table 4). These 18 effector genes are possibly the core effector set for *Xcc* that cause black rot. Additionally, the effector repertoire is highly conserved in the BJSJQ20200612 and HRIW 3811 genomes except for *xopJ*. It is noteworthy that *xopR*, *xopH*, *avrBs1*, and *xopAH* are identified in ATCC 33913, *Xcc* 8004, B100, HRIW 3811, and BJSJQ20200612 genomes, but they are absent in the GSXT20191014 genome (Table 4).

**TABLE 4:** Effector repertoire of ATCC 33913, *Xcc* 8004, B100, HRIW 3811, BJSJQ20200612 and GSXT20191014 genomes.

Effector class	Gene ID in ATCC 33913	Gene ID in *Xcc* 8004	Gene ID in B100	Gene position in HRIW 3811	Gene ID in BJSJQ 20200612	Gene ID in GSXT 20191014	Function/features
*avrBs1*	XCC2100	XC 2081	XCCB100 2396	2,790,169–2,791,505	GE002101	None	Unknown
*avrBs2*	XCC0052	XC 0052	XCCB100 0057	65,571–67,727	GE000051	GE000786	Glycerophosphoryl diester Phosphodie sterase
*avrXccA1*	XCC4229	XC 4318	XCCB100 4450	5,049,656–5,051,527	GE004369	GE000215	Unknown, maybe not a t3e
*avrXccA2*	XCC2396	XC 1716	XCCB100 1770	2,046,485–2,048,172	GE001720	GE001955	Unknown, maybe not a t3e
*hpaA*	XCC1224	XC 3018	XCCB100 3081	3,509,796–3,510,611	GE003035	GE003115	TypeIII secretion control protein, maybe not a t3e
*hpaW*	XCC1219	XC 3023	XCCB100 3086	3,512,976–3,513,965	GE003040	GE003120	Pectate lyase, maybe not a t3e
*xopA*	XCC1240	XC 3002	XCCB100 3065	3,495,949–3,496,314	GE003019	GE003099	Harpin,maybe not a t3e
*xopAC*	XCC2565	XC 1553	XCCB100 1596	1,832,580–1,834,190	GE001543	GE001790	LRR-Fic/DOC protein;vascular hypersensitivity in Arabidopsis landrace Col-0
*xopAG*	XCC3600	XC 0563	XCCB100 0580	653,171–654,430	GE000554	GE000805	Unknown
*xopAH*	XCC2109	XC 2004	XCCB100 2071	2,402,835–2,403,830	GE002018	none	Unknown
*xopAL*	XCC1246	XC2995	XCCB100 3058	3,490,465–3,491,463	GE003013	GE003094	Unknown
*xopAL*	XCC3574	XC 3916	XCCB100 0616	None	None	None	Unknown
*xopAM*	XCC1089	XC 3160	XCCB100 3256	3,713,707–3,714,666	GE003204	GE003256	Unknown
*xopAY*	XCC1073	XC3176	XCCB100 3273	3,734,293–3,735,042	GE003220	GE003272	Unknown
*xopAZ*	XCC1310	XC 2929	None	3,415,570–3,416,043	GE002947	GE003026	SlpA superfamily FKBP-type peptidylprolyl cis-trans isomerase
*xopD*	XCC2896Ψ	XC 1213Ψ	XCCB100 1256	None	None	None	C48-family SUMO cysteine protease (UIp1 protease family) (Clan CE); EAR motif; DNA binding; nuclear localization
*xopE*	XCC1629	XC 2602	None	None	None	None	Putative transglutaminase
*xopF*	XCC1218Ψ	XC 3024Ψ	XCCB100 3087Ψ	3,514,335–3,515,202	GE003041	GE003121	Unknown
*xopG*	XCC3258	XC 0967Ψ	XCCB100 2655	None	None	None	M27-family. Peptidase (Clostridium toxin)
*xopH*	XCC2099	XC 2082	XCCB100 2395	2,789,763–2,790,077	GE002102	None	Putative tyrosine phosphatase
*xopJ*	XCC3731	XC 3802	None	None	GE003851	None	Putative C55-family cysteine protease or Ser/Thr acetyltransferase(Clan CE)
*xopK*	XCC2899	XC 1210	XCCB100 1254	1,441,080–1,443,419	GE001204	GE001453	Unknown
*xopL*	XCC4186	XC 4273	XCCB100 4400	4,990,156–4,991,670	GE004323	GE000172	LRR protein
*xopM*	XCC1242	XC 3000	None	3,494,760–3,494,921	GE003017	GE003097	Unknown
	XCC1243	XC 2999	None	3,494,230–3,494,763	GE003016	GE003096	Unknown
	XCC1249	XC 2992	XCCB100 3062	3,486,174 truncated	3,528,278 truncated	GE003090	Unknown
*xopN*	XCC0231	XC 0241	XCCB100 0241	283,597–285,756	GE000240	GE000499	ARM/HEAT repeat
*xopP*	XCC1247	XC 2994	XCCB100 3057	3,487,706–3,489,907	GE003012	GE003092	Unknown
*xopQ*	XCC1072	XC 3177	XCCB100 3274	3,735,481–3,736,860	GE003221	GE003273	Putative inosine uridine nucleoside N-ribohydrolase
*xopR*	XCC0258	XC 0268	XCCB100 0280	316,317–317,552	GE000267	None	Unknown
*xopX*	XCC0529	XC 0541	XCCB100 0558	None	GE000530	None	Unknown
	XCC0530	XC 0542	XCCB100 0559	624,606–625,445	None	GE000786	Unknown
*xopZ*	XCC1975	XC 2210	XCCB100 2274	2,633,824–2,637,990	GE002235	GE002414	Unknown

*None represents that there is no homolog in this genome, truncated denotes that a truncated homolog in the genome, Ψ indicates that the homologs appear to be pseudogenes.*

Taken together, the significant differences in the composition of above mentioned virulence factors may lead to GSXT20191014 isolate infections being more aggressive in cabbage inbred line 1371.

## Discussion

We sequenced the complete genome of BJSJQ20200612 and GSXT20191014 and compared them with HRIW 3811, B100, CFBP 1869, ATCC 33913, CFBP 5817, and *Xcc* 8004. Our results showed that large-scale genomic rearrangements were identified in both strains. These two genomes are quite different in the number of predicted CDSs, variable genomic regions, virulence factors (adhesion, secreted proteins, type III effectors, and the composition of EPS and LPS) (Tables 1–4 and Figure 8). These differences may contribute to the genomic sequence and virulence variations observed in *Xcc* strains.

It has been reported that genomic rearrangements of *Xanthomonas* genomes are often due to IS and transposons, which are responsible for bacterial pathogenicity and evolution (Ferreira et al., 2015). In this study, the pathogenicity of the GSXT20191014 isolate was more virulent than HRIW 3811 in cabbage inbred line 1371 and other brassica crops. By focusing on these variable genomic regions, we found that there were 26 and 47 variable genomic regions in the genomic sequence of BJSJQ2020061 and GSXT20191014, respectively (Supplementary Table 5). Most of the variable genomic regions possess IS family transposases, which are located at each end of insertion sequence and involved in the transposition of the insertion sequence, indicating that these DNA sequences may have been acquired through horizontal gene transfer (Garcia-Vallve et al., 2003; Ou et al., 2006; He et al., 2007). The horizontal gene transfer events that occurred in the genomic sequence of the GSXT20191014 were greater in scale than that in BJSJQ2020061.

Multiple strain-specific proteins were identified through comparative analysis of variable genomic regions in BJSJQ2020061 and GSXT20191014 genomes, especially virulence factors (adhesin, secreted proteins and Type III effectors) that may contribute to the differences in virulence. For example, the gene GE000326 encoding a type IV secretion system attachment mediating protein VirB1 homolog was found in the variable genomic regions of the GSXT20191014 genome (Table 2). VirB1 was reported to promote T-pilus assembly involving the attachment of *Agrobacterium tumefaciens* (Zupan et al., 2007). Here, GE000326 encoding VirB1 homolog was found in the variable genomic region may increase the attachment and virulence of GSXT20191014. The gene GE002595 (*fhaB*) encoding FHA proteins were specifically identified at the variable genomic regions of the GSXT20191014 genome. FHA, as the major adhesin of pathogens, is one of the most efficiently secreted proteins by means of the TPS pathway in Gram-negative bacteria, which is responsible for bacterial adherence (Clantin et al., 2004). Furthermore, it is still worth emphasizing that among the GSXT20191014 strain-specific genes, two copies of peptidase S8/S53 were found in the variable genomic regions of the GSXT20191014 genome (Table 3). Secreted proteins LtSP13 from *Lasiodiplodia theobromae* and Mp1 protein from *Magnaporthiopsis poae* contain conserved peptidase S8 domain known to be involved in plant pathogenesis (Sreedhar et al., 1999; Li Z. et al., 2019). Hence, it is possible that the secreted peptidase S8/S53 could be involved in strategies for establishing infection in cabbage. It was noteworthy that so many virulence factors with different functions were found at sites 1, 16, 26, and 33 of the variable genome regions in the GSXT20191014 genome (Tables 2, 3), meaning horizontal gene transfer introduced more pathogenic-related proteins in GSXT20191014. Taken together, many insertion events have occurred in the genome of GSXT20191014 during its evolution, which eventually made this segment of genome unique in GSXT20191014 and generated the variability in adhesion, secreted proteins and effector repertoires compared to other *Xcc* strains. And all these factors could contribute to the high pathogenicity of GSXT20191014.

Previous research showed that *Xcc* strains exhibit diversity in the physiological races even in the same subspecies (Vicente et al., 2001). The release of *Xcc* genomic data (Ribeiro da Silva et al., 2002; Qian et al., 2005; Vorhölter et al., 2008; Bolot et al., 2015), has led to the development of molecular markers from DNA polymorphic regions. Nou’s group in Korea have developed specific markers for *Xcc* strains HRIW 3811, HRIW 3849A, HRIW 5212, HRIW 1279A, and HRIW 3880, according to whole genome alignments, which could work more quickly, efficiently and reliably in the detection of specific *Xcc* strains (Afrin et al., 2018b,^2019^; Rubel et al., 2017, 2019b). Here, we also developed the specific molecular makers for BJSJQ20200612 and GSXT20191014, respectively, based on the variable genomic regions (Figure 6). These markers work efficiently in distinguishing the BJSJQ20200612 and GSXT20191014 genetic subgroups from other *Xcc* strains, revealing that the diversity and distribution of *Xcc* subgroups in the main cabbages-growing areas of China. The subgrouping results are consistent with that generated by MLST method. In addition, our findings suggest that the isolates from the same subgroups may have many similarities in disease aggressiveness in cabbage inbred line 1371, so accurate identification and recognition of *Xcc* in cabbages could help breeders rapidly distinguish the *Xcc* subgroups, aiding effective control measures.

In this study, three genetic subgroups of *Xcc* were found in our isolates (Figures 3, 5, 7). Notably, we obtained two race 1 isolates (GDGZ20191212 and ZJHZ20191121) from coastal provinces Guangdong and Zhejiang, which are in the south of China. Interestingly, HBCL20191115, SXXY20191115, and GSXT20191014 belonging to the subgroup III exhibited higher virulence than HRIW 3811 in cabbage inbred line 1371 and other brassica crops (Figure 2 and Supplementary Figure 1). Isolates SXXY20191115 and GSXT20191014 were found in the cabbages grown in northwest Shaanxi and Gansu provinces; and another isolate belongs to the same genetic subgroup was obtained from Changli County of the Hebei province. The rest of the isolates belonged to the genetic subgroup II strain, which was the dominant strain that has probably evolved from race 1 (Figure 7). We speculate that the *Xcc* are rapidly evolving as ISs were identified in the genomic sequences, which usually leads to rearrangement of genomic sequence and generation of more virulent strains.

At last, accurate identification and recognition of *Xcc* in cabbage could help breeders rapidly distinguish the *Xcc* subgroups, is a prerequisite in taking effective control measures. Ill-Sup Nou’s group found that the existence of race-specific resistance to *Xcc* races B100, HRIW 3849A, HRIW 5212, HRIW 3880, HRIW 6181, and HRIW 8450A in the cabbage germplasms (Afrin et al., 2018a). They also identified markers linked to *Xcc* races HRIW 6181 and HRIW 8450A resistance in cabbage (Hong et al., 2021). It seems that the broad spectrums of disease-resistant cabbage materials were shortage, the subgroup-specific resource could be found. So it was important to know if the isolates were from a group or another.

## Conclusion

In conclusion, this is the first extensive analysis of the genetic diversity and distribution of *Xcc* based on whole genomic variation of *Xcc* strains in main cabbage growing areas of China, which could provide insight into for black rot-resistance breeding. The most effective way to control black rot wilt in brassica crops is to breed resistant varieties. In this study, our results showed that BJSJQ20200612 and GSXT20191014 genomes vary in the number of predicted CDSs, variable genomic regions, virulence factors (adhesion, secreted proteins, type III effectors, and the composition of EPS and LPS). These differences may contribute to the genomic sequence and virulence variations observed in *Xcc* strains. Meanwhile, our isolated *Xcc* strains were classified into three genetic subgroups according to MLST and our developed specific markers. However, it is important to notice that the pathogenicity experiment was done in controlled conditions and if conditions vary, the results might vary as well. In addition, there was only one cabbage inbred line 1371 tested and this might not be the case for all other cabbage lines. In the future, we aim to obtain more isolates to confirm the distribution and the virulence of *Xcc* in China. The pathogenicity test will be done in more cabbage inbred lines. At last, we aim to use these identified strains to screen and generate *Xcc-*resistant cabbage cultivars.

## Data Availability Statement

The datasets presented in this study can be found in online repositories. The names of the repository/repositories and accession number(s) can be found in the article/Supplementary Material.

## Author Contributions

XZ, JX, and JK conceived, designed the experiments, and revised the manuscript. DC, JC, and HL performed the experiments. RH and XY analyzed the data. DC and XZ wrote the manuscript. All the authors have read and approved the final manuscript.

## Conflict of Interest

The authors declare that the research was conducted in the absence of any commercial or financial relationships that could be construed as a potential conflict of interest.

## Publisher’s Note

All claims expressed in this article are solely those of the authors and do not necessarily represent those of their affiliated organizations, or those of the publisher, the editors and the reviewers. Any product that may be evaluated in this article, or claim that may be made by its manufacturer, is not guaranteed or endorsed by the publisher.

## Footnotes

1
https://www.ncbi.nlm.nih.gov/COG/

2
http://www.genome.jp/kegg/

3
http://www.mgc.ac.cn/VFs/

4
http://xanthomonas.org/t3e.html
