# Supplementary material for: Comparative Genomic Analysis of Xanthomonas campestris pv. campestris Isolates BJSJQ20200612 and GSXT20191014 Provides Novel Insights Into Their Genetic Variability and Virulence
Source: Front Microbiol. 2022 Mar 2;13:833318. doi: 10.3389/fmicb.2022.833318 (PMC8924526; doi:10.3389/fmicb.2022.833318)
Supplement: Supplementary file 1 [file Image_1.PDF]

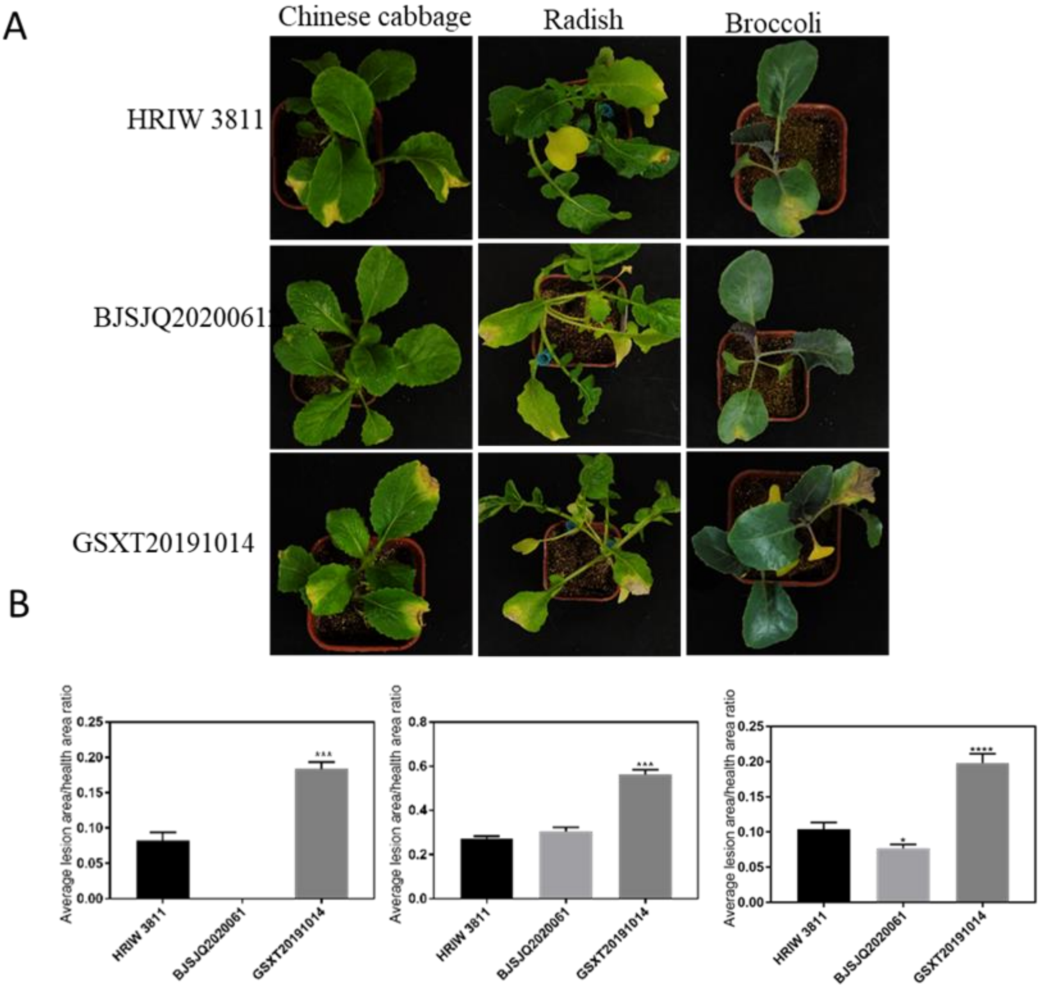

Supplementary Figure 1 | Pathogenicity analysis of HRIW 3811, BJSJQ20200612 and GSXT20191014 on Chinese cabbage, radish and broccoli. (A) All three *Xcc* strains caused typical V-shaped lesions in the Chinese cabbage, radish and broccoli after leaf clipping inoculation. **(B)** The average lesion area/health area ratio triggered by the GSXT20191014 were bigger than that caused by HRIW 3811 and BJSJQ20200612. Lesion and health area were measured using 20 leaves samples at 10 d post-inoculation (Data are means ± SD; $n > 3$; *$P < 0.05$ and **$P<0.01$).
