# Supplementary material for: Comparative Genomic Analysis of Xanthomonas campestris pv. campestris Isolates BJSJQ20200612 and GSXT20191014 Provides Novel Insights Into Their Genetic Variability and Virulence
Source: Front Microbiol. 2022 Mar 2;13:833318. doi: 10.3389/fmicb.2022.833318 (PMC8924526; doi:10.3389/fmicb.2022.833318)
Supplement: Supplementary file 2 [file Image_2.PDF]

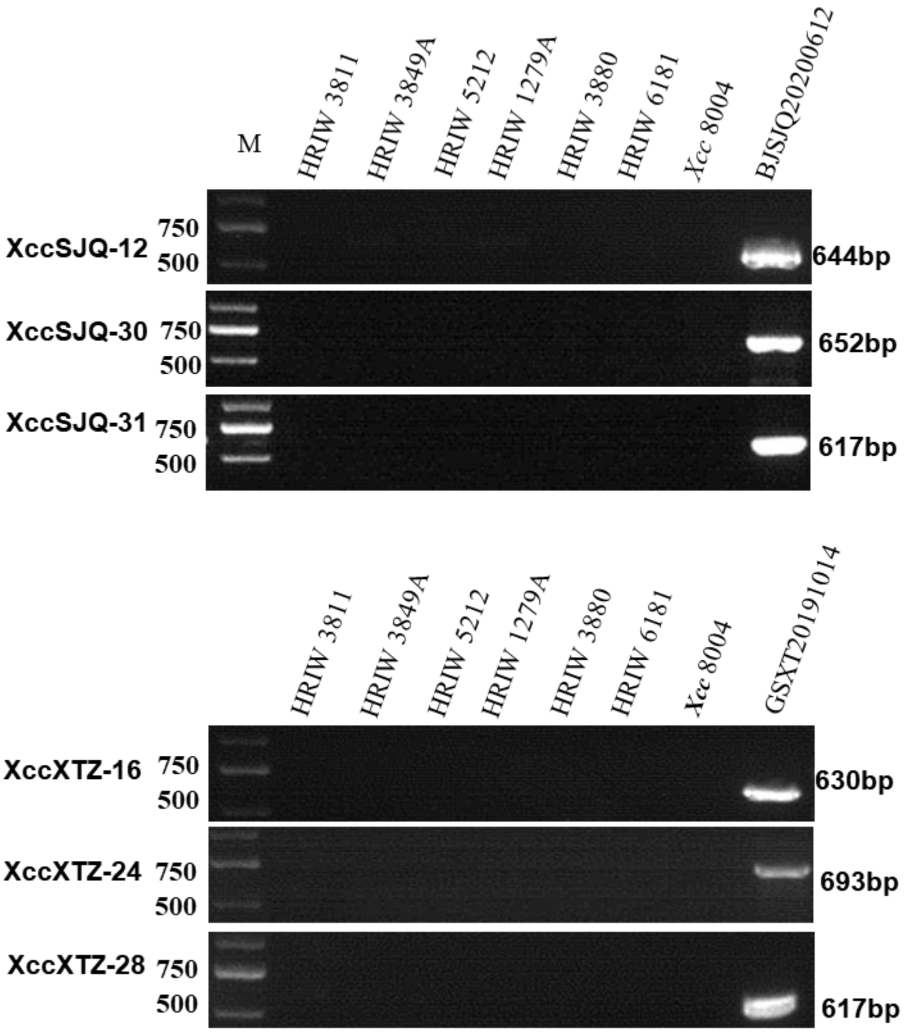

Supplementary Figure 2 | PCR amplification of Xcc strains (HRIW 3811, HRIW 3849A, HRIW 5212, HRIW 1279A, HRIW 3880, HRIW 6181 and Xcc 8004). The usability of molecular markers XccSJQ-12, XccSJQ-30, XccSJQ-31, XccXTZ-16, XccXTZ-24, XccXTZ-28 were tested by PCR using the genomic DNA of *Xcc* strains (HRIW 3811, HRIW 3849A, HRIW 5212, HRIW 1279A, HRIW 3880, HRIW 6181 and *Xcc* 8004). DNA concentration of all samples was 50 ng/μl. Lane M: 2k plus DNA ladder was used as the size marker. BJSJQ20200612 and GSXT20191014 were used as the positive control on the upper and lower panel, respectively.
