# Supplementary material for: Comparative Genomic Analysis of Xanthomonas campestris pv. campestris Isolates BJSJQ20200612 and GSXT20191014 Provides Novel Insights Into Their Genetic Variability and Virulence
Source: Front Microbiol. 2022 Mar 2;13:833318. doi: 10.3389/fmicb.2022.833318 (PMC8924526; doi:10.3389/fmicb.2022.833318)
Supplement: Supplementary file 3 [file Table_1.DOC]

**Supplementary Table 1. List of *Xcc* pathovars-specific primers used in this study.**

|  | Primers | Sequences  (5’-3’) | Product length | Annealing Temperature and reaction cycles | Reference |
| --- | --- | --- | --- | --- | --- |
| 1 | Xcc48F | CGGTGCCAGCGACTCGCCACG | 855 bp | 70°C for 40 sec, 20 cycle | Rubel et al (2019) |
|  | Xcc48R | TCCACGGCGGCGGGCCGATCTG |  |  |  |
| 2 | Xcc53F | CGTTACACGGGTCTGGAGAA | 930 bp | 60°C for 40 sec, 25 cycle | Rubel et al (2019) |
|  | Xcc53R | CCACCTGAAACTGGACGTG |  |  |  |
