# Supplementary material for: Comparative Genomic Analysis of Xanthomonas campestris pv. campestris Isolates BJSJQ20200612 and GSXT20191014 Provides Novel Insights Into Their Genetic Variability and Virulence
Source: Front Microbiol. 2022 Mar 2;13:833318. doi: 10.3389/fmicb.2022.833318 (PMC8924526; doi:10.3389/fmicb.2022.833318)
Supplement: Supplementary file 4 [file Table_2.DOC]

**Supplementary Table 2. List of BJSJQ20200612 and GSXT20191014-specific primers used in this study.**

| isolates | Primer | Sequence(5’-3’) | Product length | Annealing  Temperature |
| --- | --- | --- | --- | --- |
| BJSJQ20200612 | XccSJQ-12-F | CTCGGATAGGTCTTTTGGCG | 644 bp | 65℃, 30s |
|  | XccSJQ-12-F | CGCTACAGCGCCATACAAGT |  |  |
|  | XccSJQ-30-F | ATGAAGATCGCAAGGATTGC | 652 bp | 65℃, 30s |
|  | XccSJQ-30-R | TCTGGAGGAATCCCGAGACC |  |  |
|  | XccSJQ-31-F | CAAGTCTGGCAATCGCTTGA | 617 bp | 65℃, 30s |
|  | XccSJQ-31-R | TCCTTTGCAAGGCGACTCTC |  |  |
|  | XccSJQ-62 -F | CGTCGAGATGGCGATCTGGC | 1512 bp | 67℃, 30s |
|  | XccSJQ-62 -R | GCCGGTCGAGCAACTCGAGT |  |  |
| GSXT20191014 | XccXTZ-10-F | GGCAGCGCGCTTGATCCTTC | 510 bp | 70℃, 30s |
|  | XccXTZ-10-R | GGTCTGAGACATCGTCCGCCG |  |  |
|  | XccXTZ-16-F | TGGAGTAAGCGTCCTGCGAG | 630 bp | 70℃, 30s |
|  | XccXTZ-16-R | GATCCGCTCACGCTTGAGTA |  |  |
|  | XccXTZ-24-F | GGTCTTGGCAACATCCTGCA | 693 bp | 70℃, 30s |
|  | XccXTZ-24-R | CGCAGGCTGTTCGTAGAACA |  |  |
|  | XccXTZ-28-F | GGTTGTTGCCTGCCTGCAGT | 617 bp | 70℃, 30s |
|  | XccXTZ-28-R | CTACCAGGGCCAGAAGGTGC |  |  |
