# Supplementary material for: Comparative Genomic Analysis of Xanthomonas campestris pv. campestris Isolates BJSJQ20200612 and GSXT20191014 Provides Novel Insights Into Their Genetic Variability and Virulence
Source: Front Microbiol. 2022 Mar 2;13:833318. doi: 10.3389/fmicb.2022.833318 (PMC8924526; doi:10.3389/fmicb.2022.833318)
Supplement: Supplementary file 5 [file Table_3.DOC]

**Supplementary Table 3. Genetic diversity of 24 strains by MLSA.**

| Gene | Fragment size (bp) | No. of sequences selected | No. of alleles | No. of polymorphic sites | % of polymorphic sites |
| --- | --- | --- | --- | --- | --- |
| *atpD* | 648 | 24 | 6 | 23 | 3.5% |
| *fyuA* | 771 | 24 | 6 | 20 | 2.6% |
| *gyrB* | 705 | 24 | 5 | 13 | 1.8% |
| *rpoD* | 807 | 24 | 5 | 51 | 6.3% |
| Concatenated | 2931 | 24 | 8 | 107 | 3.6% |
