# Supplementary material for: Comparative Genomic Analysis of Xanthomonas campestris pv. campestris Isolates BJSJQ20200612 and GSXT20191014 Provides Novel Insights Into Their Genetic Variability and Virulence
Source: Front Microbiol. 2022 Mar 2;13:833318. doi: 10.3389/fmicb.2022.833318 (PMC8924526; doi:10.3389/fmicb.2022.833318)
Supplement: Supplementary file 6 [file Table_4.DOC]

**Supplementary Table 4. Number of genes associated with general COG functional categories of BJSJQ20200612 and GSXT0191014.**

| Category | Code | Value | | % age | | Description |
| --- | --- | --- | --- | --- | --- | --- |
|  |  | BJSJQ20200612 | GSXT20191014 | BJSJQ20200612 | GSXT20191014 |  |
| Metabolism | C | 184 | 183 | 4.97 | 5.09 | Energy production and conversion |
|  | E | 235 | 232 | 6.35 | 6.45 | Amino acid transport and metabolism |
|  | F | 62 | 62 | 1.68 | 1.72 | Nucleotide transport and metabolism |
|  | G | 210 | 214 | 5.68 | 5.95 | Carbohydrate transport and metabolism |
|  | H | 104 | 105 | 2.81 | 2.92 | Coenzyme transport and metabolism |
|  | I | 123 | 124 | 3.32 | 3.45 | Lipid transport and metabolism |
|  | P | 213 | 210 | 5.76 | 5.84 | Inorganic ion transport and metabolism |
|  | Q | 62 | 62 | 1.68 | 1.72 | Secondary metabolites biosynthesis, transport and catabolism |
| Cellular processes and signaling | D | 29 | 30 | 0.78 | 0.83 | Cell cycle control, cell division, chromosome partitioning |
|  | M | 216 | 210 | 5.84 | 5.84 | Cell wall/membrane/envelope biogenesis |
|  | N | 82 | 78 | 2.22 | 2.17 | Cell motility |
|  | O | 156 | 157 | 4.22 | 4.36 | Posttranslational modification, protein turnover, chaperones |
|  | T | 221 | 219 | 5.97 | 6.09 | Signal transduction mechanisms |
|  | U | 92 | 95 | 2.49 | 2.64 | Intracellular trafficking, secretion, and vesicular transport |
|  | V | 56 | 55 | 1.51 | 1.53 | Defense mechanisms |
|  | W | 1 | 1 | 0.03 | 0.03 | Extracellular structures |
|  | Y | 0 | 0 | 0 | 0 | Nuclear structure |
|  | Z | 0 | 0 | 0 | 0 | Cytoskeleton |
| Information storage and processing | J | 164 | 163 | 4.43 | 4.53 | Translation, ribosomal structure and biogenesis |
|  | A | 1 | 1 | 0.03 | 0.03 | RNA processing and modification |
|  | B | 1 | 1 | 0.03 | 0.03 | Chromatin structure and dynamics |
|  | K | 179 | 181 | 4.84 | 5.03 | Transcription |
|  | L | 275 | 261 | 7.43 | 7.25 | Replication, recombination and repair |
| Poorly characterized | R | 304 | 299 | 8.22 | 8.31 | General function prediction only |
|  | S | 730 | 655 | 19.73 | 18.2 | Function unknown |
|  |  |  |  |  |  | Not in COGs |
