# Supplementary figures and images for: Comparative Genomic Analysis of Xanthomonas campestris pv. campestris Isolates BJSJQ20200612 and GSXT20191014 Provides Novel Insights Into Their Genetic Variability and Virulence
Source: Front Microbiol. 2022 Mar 2;13:833318. doi: 10.3389/fmicb.2022.833318 (PMC8924526; doi:10.3389/fmicb.2022.833318)

**Supplementary Table 5. The variable genomic regions in BJSJQ20200612 and GSXT20191014 genomes.**


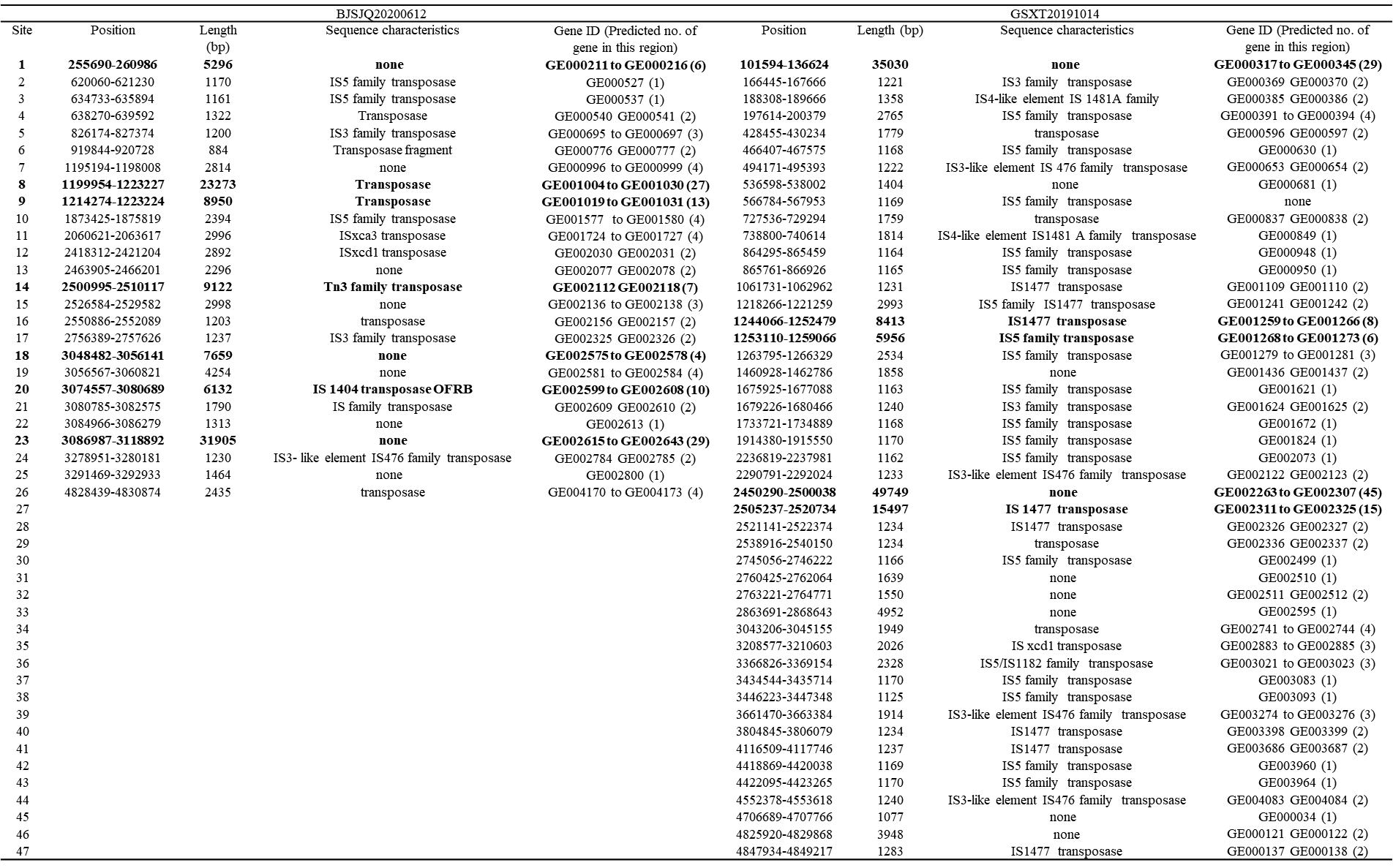

Supplement: Supplementary file 7 [file Table_5.DOC]
