# Supplementary material for: Comparative Genomic Analysis of Xanthomonas campestris pv. campestris Isolates BJSJQ20200612 and GSXT20191014 Provides Novel Insights Into Their Genetic Variability and Virulence
Source: Front Microbiol. 2022 Mar 2;13:833318. doi: 10.3389/fmicb.2022.833318 (PMC8924526; doi:10.3389/fmicb.2022.833318)
Supplement: Supplementary file 10 [file Table_8.DOC]

**Supplementary Table 8. Prediction results of signal peptide, transmembrane protein and secreted protein.**

| Protein type | BJSJQ20200612 | GSXT20191014 |
| --- | --- | --- |
| Signal peptide | 665 | 632 |
| Transmembrane protein | 1011 | 966 |
| Secreted protein | 665 | 632 |

Note: The number of secreted proteins shown here is underestimated. The type III effectors were not included.
