# Supplementary material for: Comparative Genomic Analysis of Xanthomonas campestris pv. campestris Isolates BJSJQ20200612 and GSXT20191014 Provides Novel Insights Into Their Genetic Variability and Virulence
Source: Front Microbiol. 2022 Mar 2;13:833318. doi: 10.3389/fmicb.2022.833318 (PMC8924526; doi:10.3389/fmicb.2022.833318)
Supplement: Supplementary file 11 [file Table_9.DOC]

**Supplementary Table 9. The homology of T3SS gene cluster in the *Xcc* 8004, HRIW 3811, BJSJQ20200612 and GSXT20191014 genomes.**

|  | Genes in *Xcc* 8004 | Genes in HRIW 3811 | Genes in BJSJQ20200612 | Genes in GSXT20191014 |
| --- | --- | --- | --- | --- |
| 1 | *hpa2* | 100 | 100 | 99.76 |
| 2 | *hapa1* | 99.73 | 99.73 | 100 |
| 3 | *hrcC* | 100 | 100 | 100 |
| 4 | *hrcT* | 99.88 | 100 | 99.88 |
| 5 | *hrpB7* | 100 | 100 | 100 |
| 6 | *hrcN* | 99.62 | 99.62 | 99.92 |
| 7 | *hrpB5* | 99.86 | 99.86 | 99.86 |
| 8 | *hrpB4* | 100 | 100 | 100 |
| 9 | *hrcJ* | 99.87 | 100 | 99.87 |
| 10 | *hrpB2* | 99.75 | 100 | 100 |
| 11 | *hrpB1* | 100 | 100 | 100 |
| 12 | *hrcU* | 99.81 | 99.81 | 99.91 |
| 13 | *hrcV* | 99.79 | 100 | 100 |
| 14 | *hrpaP* | 99.84 | 100 | 99.84 |
| 15 | *hrcQ* | 100 | 99.78 | 99.78 |
| 16 | *hrcR* | 100 | 100 | 99.84 |
| 17 | *hrcS* | 100 | 100 | 100 |
| 18 | *hpaA* | 99.88 | 100 | 99.88 |
| 19 | *hrpD5* | 100 | 100 | 100 |
| 20 | *hrpD6* | 99.18 | 99.59 | 99.59 |
| 21 | *hrpE* | 100 | 99.27 | 100 |
| 22 | *hpaB* | 99.79 | 99.79 | 99.79 |
| 23 | *hrpW* | 99.79 | 99.79 | 100 |
| 24 | hypothetical protein | 99.77 | 99.88 | 99.77 |
| 25 | *hrpF* | 99.89 | 99.89 | 99.71 |
